# Evaluating a Virtual Reality Game to Enhance Teen Distracted Driving Education: Mixed Methods Pilot Study

**DOI:** 10.2196/60674

**Published:** 2024-11-26

**Authors:** Colleen M Peterson, Timothy Visclosky, Carol A Flannagan, Prashant Mahajan, Andrew Gabanyicz, Jean-Jacques Bouchard, Vincent Cervantes, William Gribbin, Andrew Nobuhide Hashikawa

**Affiliations:** 1 Transportation Research Institute University of Michigan Ann Arbor, MI United States; 2 Michigan Medicine-Department of Emergency Medicine University of Michigan Ann Arbor, MI United States; 3 Michigan Medicine-Department of Child and Family Life Services University of Michigan Ann Arbor, MI United States; 4 Indiana University School of Medicine Indiana University Indianapolis, IN United States

**Keywords:** safety, virtual reality, VR, distracted driving, intervention, inattention, smartphone, novice drivers, risky driving, mobile phone, awareness, game, driving education, gamification, adolescent

## Abstract

**Background:**

Inexperienced adolescent drivers are particularly susceptible to engaging in distracted driving behaviors (DDBs) such as texting while driving (TWD). Traditional driver education approaches have shown limited success in reducing motor vehicle crashes among young drivers.

**Objective:**

We tested an innovative approach to help address the critical issue of DDB among teenagers. We investigated the effectiveness of using a novel virtual reality (VR) game “Distracted Navigator” to educate novice teenage drivers about DDB.

**Methods:**

The game consisted of maneuvering a spaceship around asteroids while engaging in simulated DDB (eg, inputting numbers into a keypad). A physician-facilitated discussion, based on the theory of planned behavior, linked gameplay to real-life driving. Teenagers were recruited for the in-person study and randomly assigned at the block level to intervention (VR gameplay or discussion) and control groups (discussion only), approximating a 2:1 ratio. Unblinded, bivariate statistical analyses (all 2-tailed *t* tests or chi-square tests) and regression analyses measured programming impact on TWD-related beliefs and intentions. Content analysis of focus group interviews identified thematic feedback on the programming.

**Results:**

Of the 24 participants, 15 (63%) were male; their ages ranged from 14 to 17 (mean 15.8, SD 0.92) years, and all owned cell phones. Compared to the control group (n=7, 29%), the intervention group (n=17, 71%) was more likely to report that the programming had positively changed how they felt about texting and driving (?^2^_18_=–8.3; *P*=.02). However, specific TWD attitudes and intentions were not different by treatment status. Irrespective of treatment, pre- and postintervention scores indicated reduced confidence in safely TWD (ie, perceived behavioral control; β=–.78; t_46_=–2.66; *P*=.01). Thematic analysis revealed the following: (1) the VR gameplay adeptly portrayed real-world consequences of texting and driving, (2) participants highly valued the interactive nature of the VR game and discussion, (3) both the VR game and facilitated discussion were deemed as integral and complementary components, and (4) feedback for improving the VR game and discussion.

**Conclusions:**

Our findings show that the novel use of immersive VR experiences with interactive discussions can raise awareness of DDB consequences and is a promising method to enhance driving safety education. The widespread accessibility of VR technology allows for scalable integration into driver training programs, warranting a larger, prospective, randomized study.

## Introduction

### Background

Motor vehicle crashes remain the leading cause of mortality among teenagers worldwide [1] and unintentional death in persons aged 15-24 years in the United States [2]. Young novice drivers are especially vulnerable to crashes or near crashes related to distracted driving behaviors (DDBs) such as texting while driving (TWD) because of their inexperience, poor risk assessment skills, and ubiquitous use of cell phones [3-5]. DDBs, and TWD in particular, have negative effects on driving performance through the detrimental effects of shared attention, task switching, inattentional blindness, and increased cognitive load while driving distracted [6]. To mitigate these harms, nearly all US states have implemented laws prohibiting TWD in some way, often imposing more restrictions for younger or more inexperienced drivers (ie, graduated licensing restrictions) [7].

### Teen Distracted Driving Paradox

Despite teens acknowledging the dangers of distracted driving and generally supporting laws limiting TWD [8], they also continue to engage in DDB [9,10] and are more likely to be in crashes involving distracted driving [11,12]. Phone blocking apps have been found to be more effective than a control condition in reducing TWD among teen drivers [13], but they do not eliminate it, in part because of a lack of motivation to use the apps [14-16]. Unfortunately, even if motivated, novice teen drivers often lack the ability to comply with texting bans due to poor impulse control [17] and, relatedly, problematic cell phone use [18]. These issues, combined with the perceived advantages of TWD and the perceived utility of compensatory strategies [9], may outweigh the acknowledged risks among young novice drivers who engage in DDB [19].

### Potential for Virtual Reality

The educational paradigm for teenage driver’s education has remained static, with most evaluation research demonstrating limited effectiveness in reducing crashes among young novice drivers [20], although there are recent exceptions [21]. Despite the pervasiveness of technology related to drivers’ training [22], driver’s education still relies on classroom-style textbooks, lectures, videos, and low-technology simulation (eg, a video screen connected to a simple wheel and pedal). Our goal was to develop an intervention to augment traditional in-person driver’s education.

Head-mounted display–based virtual reality (VR) is an exciting technology used for gamification and is increasingly used for learning and safety training in multiple fields [23-27]. Providing immersive, experiential evidence of how distractions adversely affect performance through VR could help address problematic teenage and novice driving behaviors [28-30]. Research involving VR and drivers training to date has featured primarily postlicensing skill assessment targeting generalized improvement [29,31], such as through hazard perception and mitigation training [28,32] and error identification [31] of adult drivers. Only Jakab [29] specifically recruited those with “having close to no experience with driving in real life.”

To the authors’ knowledge, just one early VR study by Morley et al [33] focused on distracted driving specifically. Morley et al [33] engaged a small number of participants, aged 20-40 years, in driving around a virtual track. Once familiar with the track, they were asked to interact with a virtual smartphone, which triggered a large oncoming truck accompanied by loud horn sounds. This resulted in an unavoidable crash with crash sound effects and “violent movement from the force feedback” technology. From in-session feedback, 20 (86%) out of 23 participants said that the event reinforced or changed their perceptions of the dangers of phone use while driving. No longer-term feedback on the effects of the VR experience was collected. As Morely et al [33] and other VR driving studies demonstrate, there is definite potential for VR to address TWD as part of driver’s education.

VR is specifically recognized as a promising approach to novice driver education and training that can meet the new Novice Teen Driver Education and Training Administrative Standards [34]. VR-based games have several potential advantages over traditional video or low-technology simulators as a teaching tool: (1) presence, or the idea that the user is physically present in the virtual environment; (2) embodiment, when the user feels they are genuinely inhabiting the virtual character and having their actions mirrored within the virtual environment; and (3) physicality, where the user’s actual degree of physical activity is substantially increased during gameplay, creating an increased immersive, “learning-by-doing” experience [34,35]. By experiencing the consequences of in-game distractions, young novice drivers may reevaluate their beliefs about TWD and perceived ability to text while driving and reassess their perceived evaluation of the risks they take when engaging in TWD, which affects their attitudes and intention to engage in TWD.

The primary aim of this study was to evaluate the utility of Distracted Navigator and facilitated discussion using a mixed approach in a pre- and postintervention randomized controlled trial. We sought to test the following hypotheses: (1) intervention group participants who experience the VR game will have significantly reduced intentions to engage in TWD and (2) the intervention group will have statistically significant changes in other theory of planned behavior (TPB) constructs regarding TWD. Additionally, we used open-ended and focus group interview data (1) to gain participant insight into the utility of their VR experiences and themes supporting the quantitative data and (2) to identify qualitative themes for future development and enhancements to the VR game.

## Methods

### Distracted Navigator Intervention

By having teens experience a novel VR game featuring DDB (Distracted Navigator) and engage in a facilitated educational discussion relating gameplay to real-world consequences, we sought to make more effective education on how DDB and TWD impact driving performance.

Distracted Navigator was developed iteratively with Preview Labs [36], informed by VR PLAY [37] and other game development guidelines. Using a VR head-mounted display, a player is placed virtually inside a spaceship cockpit and has control of the speed and the direction of the ship, which they navigate through an asteroid field (Multimedia Appendix 1). Distracted Navigator provides vibrational haptic feedback via the player controller when asteroids hit the spaceship with noticeable damage to the spaceship’s windshield.

After a 5-minute onboarding tutorial and practice session, the user plays their first round of the game (without any distractions) for ~5 minutes, navigating the spaceship to avoid oncoming asteroids. They receive a score depending on how successfully they avoided damage to the ship. In round 2 of the game, distractions are introduced, as the player is exposed to several “emergency” tasks. While still navigating the ship around incoming asteroids for ~5 minutes, players must manage emergency tasks including (1) typing in a specific number sequence into a keyboard, (2) looking at orbs of lights on the sides of the cockpit to reactivate the ship’s lighting system, (3) pulling a specific sequence of overhead levers, and (4) plugging devices into the console. The game ends with comparing the round 1 (without distractions) and round 2 scores (with distractions) with an illustration of a spaceship demonstrating the level of damage received during round 2 of play.

Distracted Navigator was designed specifically to (1) be immersive and fun while experiencing the performance-reducing effects of DDB, (2) not incentivize players to improve their score while engaging in DDB, and (3) leverage the familiar-to-teen experience of watching live streaming gameplay by broadcasting individual play to other group members. Collectively, Distracted Navigator tasks simulate the effects of (1) texting, (2) taking eyes off the road, (3) multitasking, and (4) plugging in devices to demonstrate the negative effects of shared attention, task switching, inattentional blindness, and increased cognitive load while driving distracted [38]. The game also features a cartoon rooster that flies across the screen during gameplay to demonstrate inattention blindness like the classic “invisible gorilla” experiment [39].

### Facilitated Discussion

Both the intervention and the control group participated in the discussion, facilitated by a content expert familiar with working with teens (author TV), which educated participants on the dangers of DDB. Importantly, the facilitated discussion was implemented using an interactive approach akin to motivational interviewing to elicit the teens’ own perspectives and to better engage them in collaborative, thoughtful discussion [40].

**Figure 1:**
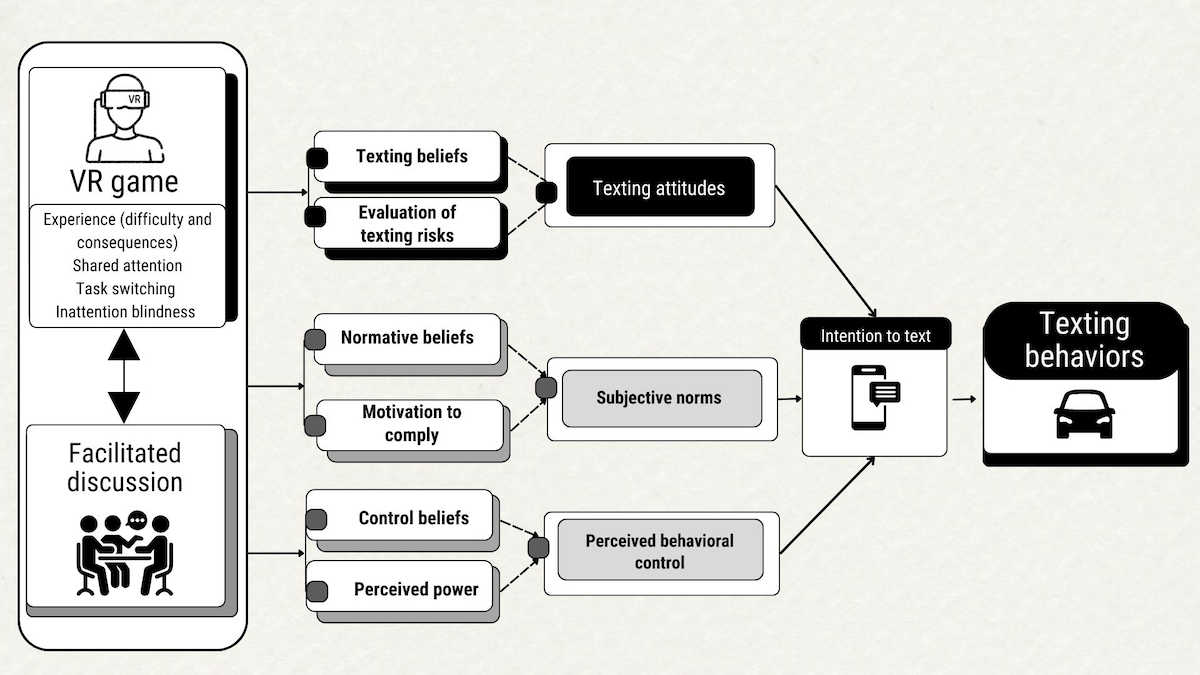
Integration of theory of planned behavior constructs within the study’s educational materials. Between the Distracted Navigator VR game and the facilitated discussion, participants explored texting attitudes, subjective norms, and perceived behavioral control related to texting while driving behaviors in an attempt to ultimately impact behaviors. VR: virtual reality.

The content was adapted from didactic educational material on distracted driving offered by the longstanding nonprofit service organization American Automobile Association (“triple A,” colloquially) [41] and the local pediatric hospital’s *DriveSmart* campaign (C.S. Mott Children’s Hospital, University of Michigan Health System Pediatric Trauma), including web-based advice to parents to support their teen’s safe driving (eg, conversation tips). The material was further enhanced by the inclusion of TPB concepts related to TWD (Figure 1).

Since the evaluation of risks, peer norms, and perceived control beliefs all play roles in the complex decision to use or not use a phone while driving [42,43], especially for young drivers [9,44,45], the TPB captures the complexity of distracted driving better than other models (eg, health belief model) [46]. The facilitator acknowledged the pressure to reply to messages while driving, and teens were asked to share whether they thought habitual phone use could make avoiding distracted driving difficult. The facilitator then elicited strategies to combat this pressure and offered other strategies, if not suggested by the teens themselves, to enhance control beliefs and perceived power. In addition, teen perceptions of multitasking ability and the utility of risk-compensation strategies were addressed. The intervention group had additional material that tied Distracted Navigator gameplay experiences to everyday driving situations and reinforced these TPB concepts.

### Participants and Procedure

Game onboarding and facilitated discussion were beta-tested with 2 teens recruited from a local children’s hospital volunteer patient advisory committee. Teenagers were recruited via email, and consent procedures were conducted with parents in a virtual meeting room. We then recruited future and novice teen drivers from a Michigan high school to pilot-test the effect of a virtual gaming intervention and accompanying discussion on TWD perceptions and attitudes from July to August 2022 and in July 2023. Authors ANH and VC liaised with a local high school to recruit teen drivers. Teens were eligible if they were aged 14-17 years. Internet and email literacy were implicit eligibility criteria. Email contact was collected for both the students and a parent or guardian. Author VC met with the teens and their parent or guardian via a virtual meeting room where eligibility was assessed, study procedures were reviewed, and written participant assent and parent or guardian consent were obtained.

**Figure 2:**
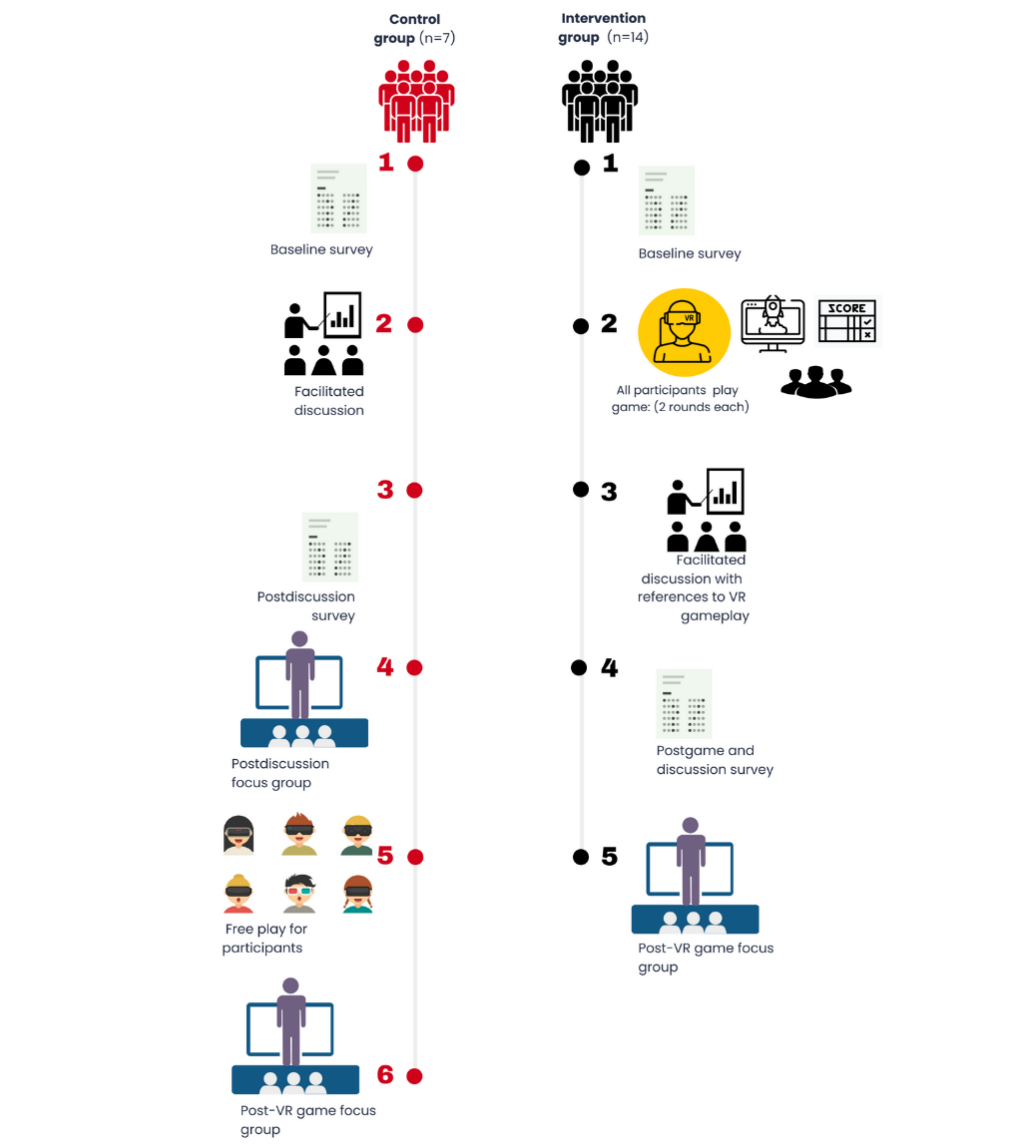
Mixed methods, pre- and postintervention, randomized controlled study design. Future and novice Michigan teen drivers were randomized at the group level to intervention or control groups in a 2:1 ratio. Following a baseline survey, intervention groups played Distracted Navigator and then participated in a facilitated discussion. They then completed a postprogram survey and engaged in a focus group on their experiences. The control group similarly completed a baseline survey before participating in a facilitated discussion without gameplay, followed by a postprogram survey and focus group. This group was then allowed a free play period with Distracted Navigator and finished by engaging in a brief additional focus group on their VR game experience. VR: virtual reality.

Teens were recruited in blocks of up to 7 and randomly assigned at the block level to intervention and control groups approximating a 2:1 ratio. All teens were asked to complete the baseline survey before the session. All other study activities took place face-to-face. In the intervention group, participants played the Distracted Navigator VR game and engaged in the facilitated driver’s education curriculum on the effects of distracted driving with references to their gameplay experiences, completed the postsession survey, and then participated in the focus group to provide additional feedback. As described earlier, the control group engaged in a similar moderator-facilitated driver’s education curriculum on the effects of distracted driving with no reference to the gameplay. They then completed the postsession feedback survey and were then allowed a free period to play the VR game for fun. Following this gameplay, the control group engaged in a second focus group session to provide feedback on the VR game. The participant study flow is shown in Figure 2. Author AG assisted in fitting study participants with VR headsets and gameplay training.

All study staff and investigators completed the University of Michigan Minors as research participants and followed the participant contact protocols. The CONSORT (Consolidated Standards of Reporting Trials)-EHEALTH checklist [47] is available in Multimedia Appendix 2.

### Measures

#### Overview

The presession (baseline) survey was emailed to the students to complete on the Qualtrics (Qualtrics International Inc) platform 24 hours before the session. All question responses included a nonresponse or neutral option. Participants were asked to complete the baseline survey before the session began. The post–facilitated discussion survey (follow-up) was sent immediately via email after the facilitated discussion and was completed before the focus group interview (see Figure 2 for study flow). Intervention and control participants received the same survey materials referring to the “programming.” The main outcomes of interest were the TPB constructs with intervention and time (baseline and follow-up) as the primary independent predictor variables. We also assessed overall perceptions of the programming.

#### Demographics and Driving Behaviors

Demographics included sex, age, race and ethnicity, and grade in school. We assessed typical hours spent driving, seatbelt habits, level of Michigan driving license, TWD frequency (typically and past week), and whether they had experienced a variety of consequences of different severities due to TWD (eg, drifted into another lane and ran stop sign or stop light). We also asked if they used text dictation software (eg, SIRI) to text while driving and had played any VR games before.

#### Intervention Assessment Overall

The follow-up survey asked participants to assess the program they experienced overall. Specifically, they were asked “Did the program change how you feel about texting and driving?” and “Did the program change how likely you are to text and drive?” Response options for both questions were yes, maybe, or no. Questions were phrased to refer to the “program” generally so both intervention and control participants received the same questions and could be compared. An initial subset of surveys was distributed without this question included in error (n=5).

#### Texting and Driving TPB Constructs

Before and after the session, participants were asked how strongly they agreed or disagreed with statements regarding their perceived behavioral control (PBC) to text and drive, general TWD attitude, subjective norms, perceived disadvantages and advantages of not engaging in TWD, and intention to TWD in the next week. Response options ranged from strongly disagree to strongly agree (1=strongly disagree, 2=agree, 3=neither agree nor disagree, 4=disagree, and 5=strongly disagree). These TPB items were derived from Gauld et al [48], Hafetz et al [49], and Harrison [50] and identified as having sufficient reliability and validity in a large sample of teen drivers [45]. The TPB as well as the TWD consequence questions were used with permission.

#### Semistructured Focus Group Interview Questions

Semistructured prompts engaged the participants in a discussion of their general impression of the game and the facilitated discussion, their likes and dislikes of the game and its components, the main takeaways of the program, and whether they would recommend it to others. Specific questions also addressed gamification and the utility of watching others play rather than experiencing the game themselves (ie, live streaming). Focus group procedures followed Krueger and Casey’s [51] guidance, and the facilitator emphasized honesty, confidentiality, and desire for negative feedback in order to avoid social desirability biases. Focus group audio was transcribed by a Health Insurance Portability and Accountability Act–compliant, third-party company. Full script guidance is available in the “Semistructured focus group guide” section in Multimedia Appendix 3.

### Analysis Plan

#### Quantitative Analysis

We compiled descriptives and conducted randomization equivalency tests using either 2-tailed *t* tests or chi-square tests as appropriate. For TPB items and overarching constructs, we conducted pre- and postcluster analysis tests of the treatment condition with a time interaction using mixed effect hierarchical modeling with restricted maximum likelihood and the residual *df* method specified, given the continuous outcomes; group assignment and individuals (by IDs) were included as random effects. The interaction with time accounted for differences in the conditions present at baseline. Models controlled for typical texting frequency, which was differential by the treatment group (*P*<.05). Given the distribution of responses, how often teens typically read or send texts while driving was dichotomized into “never” and “ever.” Partially completed questionnaires were part of the analyzed data.

Treatment assignment was assessed with the proper degrees of freedom for a cluster randomized trial (#conditions[#groups-1]=8 df) using the lincom (linear combinations of parameters) postestimation procedure. Because the test for group-level effects (intraclass correlation coefficients) with this multilevel modeling was near 0, procedures were replicated using a standard linear regression approach to explore pre- and postintervention effects with enhanced power (degrees of freedom). All quantitative analyses were conducted by author CMP, who was not blinded to treatment assignment, using StataSE (version 18.0; StataCorp LLC).

#### Qualitative Analysis

Authors CMP, ANH, and TV implemented thematic content analysis following procedures from Braun and Clarke [52] of the survey feedback and focus group transcripts. The content analysis aimed to identify themes in the VR game and facilitated the discussion’s utility and areas for improvement. Authors independently read focus group transcripts and open-ended survey responses. Together, they reviewed commonalities and any exceptions they noted to develop codes and identify overarching themes. Themes and emblematic quotes are presented. Points of convergence and divergence between the qualitative themes and quantitative results are discussed.

### Ethical Considerations

This research project was reviewed and approved by the University of Michigan Institutional Review Board (#HUM00213233). The University of Michigan Institutional Review Board has full accreditation by the Association for the Accreditation of Human Research Protection Programs, Inc. All research staff and investigators have completed the University of Michigan–mandated training on conducting research with minors in addition to human subject protection certification. No exemptions or alterations were made to the informed consent process. Written informed consent was collected virtually using a secure instance of the SIGNNow document service. As all participants were aged 14-17 years, a separate abbreviated assent document was not used in this study. Both informed consent and assent were collected with the use of the same form. A complete informed consent and assent process were conducted via a secure Zoom (Zoom Video Communications) video call with both the adolescent and parent facilitated by a Michigan Medicine research coordinator or a study investigator. In addition to consent to conduct primary research data collection and analysis, the consent document also contained permissions to use deidentified study data for future research studies and distribution without additional consent. Participants were assigned a study number, and collected data were stripped of identifiers. Audio recordings were transcribed by the researchers, and any potentially identifying information was removed. Participants who completed all study procedures were provided 1-time compensation of a US $100 gift card and provided with food and beverages during the session as incentives. These gift cards were obtained through the University of Michigan Human Subjects Incentive Program and distributed to the participants by the investigators at study completion. Identification of the participants would not be possible through any information provided in this paper or any supporting documents. All identifying or potentially identifying information is housed and maintained by the University of Michigan and is only accessible to the research team.

## Results

### Demographics and Driving Behaviors

#### Overview

A total of 24 teen drivers were recruited and assigned to intervention (n=17, 71%) or control (n=7, 29&) across 5 groups (4 intervention groups of varying sizes and 1 large control group), approximating the 2:1 assignment target. No baseline data were available from one participant, and follow-up was missing from another participant. In total, 15 (63%) teen participants were male, and their ages averaged 15.8 (SD 0.92) years, entering 10th through 12th grades about evenly. They were primarily White (n=13, 54%) and Asian (n=6, 25%). All had their own cell phone, and 17 (71%) had played a VR game before.

Teens were also about evenly distributed by what kind of license they held, from none to level 3. In total, 14 (58%) said that they typically never send a text while driving, and another 7 (29%) said they rarely do. In addition, 14 (58%) also said they never typically read a text while driving, and 5 (21%) said they rarely did. These frequencies were unevenly distributed by treatment and intervention group. The control group members reported more typically reading texts while driving (6/7, 86% vs 4/17, 24%) and sending texts while driving (5/7, 71% vs 5/17, 29%) compared to the intervention group. Table 1 shows the full demographic and comparison details.

**Table 1:** Demographics and baseline driving behaviors of the future and novice Michigan teen driver participants comparing control and intervention groups using 2-tailed t test treatment comparisona.

					Total			Control (n=7)			Intervention (n=17)			*P* value	
**Demographics**															
	**Sex (female), n (%)**			9 (8)			4 (57)			5 (29)			.20		
	**Age (years), mean (SD)**			15.8 (0.9)			16 (0.6)			15.7 (1.0)			.25		
	**Race, n (%)**														
		American Indian or Alaska Native	1 (5)			0 (0)			1 (6)			.89			
		Asian	6 (29)			2 (40)			4 (25)			.82			
		Black or African American	1 (5)			0 (0)			1 (6)			.82			
		Hispanic origin	1 (5)			0 (0)			1 (6)			.57			
		White	13 (62)			3 (60)			10 (63)			.82			
	**Grade, n (%)**														.19
		10th	6 (25)			0 (0)			6 (35)						
		11th	10 (42)			4 (57)			6 (35)						
		12th	8 (33)			3 (43)			5 (29)						
	**Michigan license held, n (%)**														.99
		None	7 (29)			2 (29)			5 (29)						
		Level 1	7 (29)			2 (29)			5 (29)						
		Level 2	6 (25)			2 (29)			4 (24)						
		Level 3	4 (17)			1 (14)			3 (18)						
**Driving behaviors**															
	**Hours driving per week (typical), n (%)**														.79
		0	7 (29)			2 (29)			5 (29)						
		1-2	4 (17)			2 (29)			2 (12)						
		3-5	7 (29)			2 (29)			5 (29)						
		6-10	4 (17)			1 (14)			3 (18)						
		11 or more	2 (8)			0 (0)			2 (12)						
	**How often do you typically send an SMS text message while driving? n (%)**														.03
		Never	14 (58)			2 (29)			12 (71)						
		Rarely	7 (29)			5 (71)			2 (12)						
		Sometimes	2 (8)			0 (0)			2 (6)						
		Often	1 (4)			0 (0)			1 (6)						
		All the time	0 (0)			0 (0)			0 (0)						
	**Within the past week, how often did you use your cell phone to send SMS text messages while driving? n (%)**														.24
		Never	21 (88)			7 (100)			14 (82)						
		A few times	3 (13)			0 (0)			3 (18)						
		Several times	0 (0)			0 (0)			0 (0)						
		Many times	0 (0)			0 (0)			0 (0)						
	**How often do you typically read an SMS text message while driving? n (%)**														.02
		Never	14 (58)			1 (14)			13 (77)						
		Rarely	5 (21)			3 (43)			2 (12)						
		Sometimes	4 (17)			3 (43)			1 (6)						
		Often	1 (4)			0 (0)			1 (6)						
		All the time	0 (0)			0 (0)			0 (0)						
	**Within the past week, how often did you use your cell phone to read SMS text messages while driving? n (%)**														.54
		Never	20 (83)			6 (86)			14 (82)						
		A few times	2 (8)			1 (14)			1 (6)						
		Several times	2 (8)			0 (0)			2 (12)						
		Many times	0 (0)			0 (0)			0 (0)						

^a^We are missing race and ethnicity data for earlier groups. License level 1=supervised, can only drive with a parent or designated licensed adult age 21 or older; level 2=intermediate, has limits on passengers and unsupervised nighttime driving; and level 3=full, all driving privileges with no restrictions. We also explored the potential for historical confounding given Michigan’s hands-free law went into effect for all drivers in June 2023, which was in between virtual reality sessions. Analyses indicate no difference in the theory of planned behavior constructs based on the timing of sessions.

#### Intervention Assessment Overall

Recalling that some participants did not receive this question due to error, we have a smaller number of providing their overall assessment of the program they experienced. Considering the program overall, 11 (79%) out of 14 participants in the treatment group said that the VR game and facilitated discussion had changed how they felt about texting and driving and how likely they were to engage in it (Table 2). For the control group, half (2/4, 50%) said that their discussion-only program “maybe” changed how they felt about texting and driving, and the other half (2/4, 50%) said that it did not change how they felt about TWD. The control group was also split in terms of how their program changed how likely they were to text and drive, with half (2/4, 50%) saying “maybe” and half (2/4, 50%) reporting “yes” it changed how likely they were to text and drive.

**Table 2:** Postsurvey responses regarding how the program changed perceptions of texting while driving between the control and intervention groupsa.

		Control (n=4), n (%)	Intervention (n=14), n (%)	Total (n=18), n (%)
**Did the program change how you feel about texting and driving?**				
	No	2 (50)	2 (14)	4 (22)
	Maybe	2 (50)	1 (7)	3 (17)
	Yes	0 (0)	11 (79)	11 (61)
**Did the program change how likely you are to text and drive?**				
	No	0 (0)	1 (7)	1 (6)
	Maybe	2 (50)	2 (14)	4 (22)
	Yes	2 (50)	11 (79)	13 (72)

^a^The intervention group was more likely to report a change in how they felt about texting while driving following both the gameplay and facilitated discussion.

Bivariate analyses indicated that people in the intervention group were more likely to say that the intervention changed how they felt about texting and driving (𝜒^2^_18_=–8.3; *P*=.02), but this difference was no longer statistically significant when modeling controlled for “ever” texting frequency. Overall program impact on intentions was not significantly different by group in either statistical approach.

### TWD TPB Constructs

#### Overview

Full cluster mixed model analyses (including as covariates for control purposes “ever” sending and “ever” reading texts while driving) accounting for proper group randomized trial degrees of freedom showed no significant interactions between treatment assignment and time (pre-post), indicating no statistically significant effects from the trial. Higher-powered regression analyses that disregard the cluster effects, which were negligible, also showed no statistically significant interactions between time and treatment (Table S1 in Multimedia Appendix 3).

#### Exploratory Analyses

Because there were no differences by treatment group, and cluster effects were minimal, we conducted exploratory pre- and postregression analyses (still controlling for sending or reading texts while driving). As shown in Table 3, PBC item #1 (“I am confident that I could text while driving and still drive safely.”) was statistically significantly different before and after the session (β=–.78; t_46_=–2.66; *P*=.01). Relatedly, the PBC sum score, which included PBC item #2 (“It would be easy for me to text while driving in the next week.”) was also significant (β=–1.28; t_46_=–2.49; *P*=.02) but not item #2 by itself. In these same models, many TPB items were significantly associated with texting frequency but not the time factor. Table S2 in Multimedia Appendix 3 shows all outputs.

**Table 3:** Exploratory pre- and postregression models were performed to measure the programming impact on texting while driving related beliefs and intentions^a^.

				β (95% CI)		SE		*t* test (*df*=46)		*P* value
**Perceived behavioral control**										
	**I am confident that I could text while driving and still drive safely**									
		Time (follow-up)	–.78 (–1.37 to –0.19)		0.29		–2.66		.01	
		Read texts ever	–.02 (–0.81 to 0.76)		0.39		–0.06		.95	
		Send texts ever	.73 (–0.06 to 1.51)		0.39		1.86		.07	
	**It would be easy for me to text while driving in the next week**									
		Time (follow-up)	–.50 (–1.07 to 0.08)		0.29		–1.74		.09	
		Read texts ever	.49 (–0.28 to 1.25)		0.38		1.28		.21	
		Send texts ever	.74 (–0.03 to 1.5)		0.38		1.93		.06	
		Intercept	1.57 (1.09 to 2.06)		0.24		6.58		<.001	
	**Perceived behavioral control sum**									
		Time (follow-up)	–1.28 (–2.31 to –0.24)		0.51		–2.49		.02	
		Read texts ever	.46 (–0.92 to 1.84)		0.68		0.68		.50	
		Send texts ever	1.46 (0.08 to 2.84)		0.68		2.14		.04	
		Intercept	3.66 (2.79 to 4.52)		0.43		8.50		<.01	

^a^The models assessed the theory of planned behavior constructs while controlling for time (pre- and postintervention) as well as sending or reading texts while driving. The most notable change after the intervention regarded the perceived behavioral control.

### Qualitative Themes from Focus Group Interviews

#### Overview

The primary themes that emerged from the qualitative dataset were (1) the VR gameplay adeptly portrayed the real-world consequences of texting and driving, (2) participants highly valued the interactive nature of the VR game and discussion, and (3) both the VR game and facilitated discussion were deemed as integral and complementary components in cultivating awareness about the risks of DDB. Finally, participants offered (4) feedback for improving the Distracted Navigator game and facilitated discussion for further intervention development.

#### VR Gameplay Adeptly Portrayed the Real-World Consequences of Texting and Driving

The immersive Distracted Navigator gameplay achieved one of its educational aims, as participants recognized how the game’s distracting tasks and declining game performance related to real-world consequences of DDBs.

It makes more apparent with how dangerous distractions really are...I was hitting five things—five asteroids when I was texting. It reminds me of that. It took about the same amount of time of pushing the buttons as a text would take. It really shows you how you can hit so many things or cause that many accidents in such a short amount of time. It was like two seconds.

This was taken further by multiple participants who reported that the consequences experienced in the game lessened their intention to engage in TWD behaviors.

For me, I’m less likely to text now. I’m not gonna lie, I’ve text and drove before, it’s happened. I think now, seeing for example, you can see the ship on the side and see how damaged it gets. I think that also helps a lot with showing what can happen in real life scenarios.

The haptic feedback and sounds via the VR equipment also helped create an immersive world.

#### Participants Highly Valued the Interactive Nature of the VR Game and Discussion

In discussion of the Distracted Navigator in particular, participants agreed that the engaging nature of the game enhanced the educational value.

This worked for me ’cause I like to interact with things. I like being able to work with the things, and playing a game made me wanna focus more.

This was true for the facilitated discussion as well. Multiple participants favorably compared this discussion to traditional driver’s education.

I like it being more of a conversation than just a teacher or somebody showing it to you.

By comparison, our facilitated discussion based on TPB concepts was “more engaging” and “interactive.” The value of this engagement was seen and was perhaps made more prominent when the game and discussion were paired together. Participants explicitly described it as “a fun educational experience,” saying, “you were having fun at the same time while learning things.”

#### Both the VR Game and Facilitated Discussion Were Deemed as Integral and Complementary Components in Cultivating Awareness About the Risks of DDB

Finally, the participants made note of the synergy between the gameplay and facilitated discussion. While our groups discussed both favorably, the combination of the 2 was identified as necessary for maximal learning.

I think it made the connection better between the game and then the slides. I think on their own, each one would have done worse, but combined, I think it did a lot better.

There was consensus that teens should play the VR game first and then engage in the discussion because this provided context and augmented, in a timely manner, exactly what had just been experienced in the game.

#### Feedback for Improving the Distracted Navigator Game and Facilitated Discussion

Focus group feedback from teens offered several areas for modifying the VR game and discussion to be more engaging and relatable to driving risks. Nearly universally, teens said they preferred more realism in the game, especially in the tasks. For example, unlike the keypad digit-entering task, the lever-pulling task did not have a good real-world analog. Some of the distraction tasks were confusing in terms of what to do and could be simplified. For the facilitated discussion, teens recognized the utility of incorporating more compelling statistics and narratives. They also suggested more surface-level changes such as more engaging graphics.

## Discussion

### Principal Findings

Although there was no statistically significant impact on novice drivers’ DDB attitudes and intentions, our findings show success in developing an intervention to augment traditional driver’s education. Participant feedback suggests that the immersive experience of Distracted Navigator reinforced the negative effects of TWD and other DDB in ways that standard drivers’ education lectures cannot.

According to the protection motivation theory [53,54], novice drivers who have limited driving experience may have an inaccurate perception of the risks or consequences and therefore may engage in behaviors like TWD more often. Our feedback suggests that Distracted Navigator was successful in providing a safe and immersive setting for teens to experience such consequences and thus could influence the perceived risk of TWD behavior. Indeed, despite most participants reporting never experiencing the consequences of DDB (eg, ticketed or a crash), participants readily connected the “emergency tasks” to real-world behaviors and the negative effects of distracted driving.

While our focus group feedback underscored the efficacy of the combined programming—facilitated discussion and Distracted Navigator—in demonstrating the repercussions of DDB and changing participants’ intentions, the quantitative data did not show analogous statistically significant outcomes. In addition to the small sample size, there are 2 primary other explanations for this discrepancy.

The first is the possible confounding effect of the facilitated discussion in isolation. All groups, control and intervention, agreed that the facilitated discussion was superior to traditional classroom-based drivers’ education. While the synergistic effect when paired with the game was clear, the benefit of the discussion alone, thoughtfully engaging teen drivers in discussion of these behaviors and related impairments, can still heighten awareness of their dangers with or without the VR gameplay.

Second, in both the preintervention survey and the focus groups, most participants expressed their intention and self-reported habits to not text and drive. This “floor effect” allowed for little movement toward a statistically significant impact. Even so, nearly three-quarters of participants had said the program changed how likely they were to text and drive when asked outside of the TPB items.

As a pilot study, our findings show promise for Distracted Navigator as a new technology-based educational tool for novice drivers. Though our study was limited by size and the possible confounding effects of a specifically designed discussion, the feedback was positive with concrete recommendations for future improvements, highlighting potential areas for future research. Another potential benefit of the VR approach is the capability to live stream VR gameplay, a popular and familiar format for teens to engage in the play by proxy, enhancing the scalability of the intervention [55,56]. Larger, longitudinal studies with greater statistical power are warranted to evaluate the potential for VR to have a lasting impact on driver’s education.

### Limitations and Strengths

Our study was constrained by the small pilot sample size, which limited statistical power and contributed to the imbalance in the baseline differences in important TWD behaviors. The study relied on self-reported data for texting behavior, which could have been susceptible to demand characteristics, and did not include a longer-term follow-up to evaluate the lasting effects of the intervention. Most participants had prior experience with VR, and all had their own cell phones, so results may not be generalizable to the teenagers living in underserved areas without exposure to technology or access to cell phones. However, we captured candid feedback that offered valuable insights into participant experiences with the VR game and facilitated discussion to inform the intervention and VR game development.

### Conclusions

The growing availability of VR systems enables the easy integration of educational VR games like Distracted Navigator to enhance driver training education programs. Insights gained from participant feedback will be used to improve the VR game and the facilitated discussion material in preparation for a larger, prospective, randomized controlled trial to evaluate their impact on DDB beliefs and objective DDBs using smartphone technologies.

## Appendix

Multimedia Appendix 1 The images represent (A) navigating around asteroids, (B) task switching by pulling levers, (C) multitasking by inputting numbers into a keypad, and (D) inattention blindness when a rooster flies across space.

Multimedia Appendix 2 CONSORT (Consolidated Standards of Reporting Trials) EHealth checklist.

Multimedia Appendix 3 Supplementary content.

## Abbreviations

CONSORT: Consolidated Standards of Reporting Trials
DDB: distracted driving behavior
PBC: perceived behavioral control
TPB: theory of planned behavior
TWD: texting while driving
VR: virtual reality

## Data Availability

The datasets generated and analyzed during this study are available from the corresponding author on reasonable request.
