# Supplementary figures and images for: Evaluating a Virtual Reality Game to Enhance Teen Distracted Driving Education: Mixed Methods Pilot Study
Source: JMIR Form Res. 2024 Nov 26;8:e60674. doi: 10.2196/60674 (PMC11632282; doi:10.2196/60674)

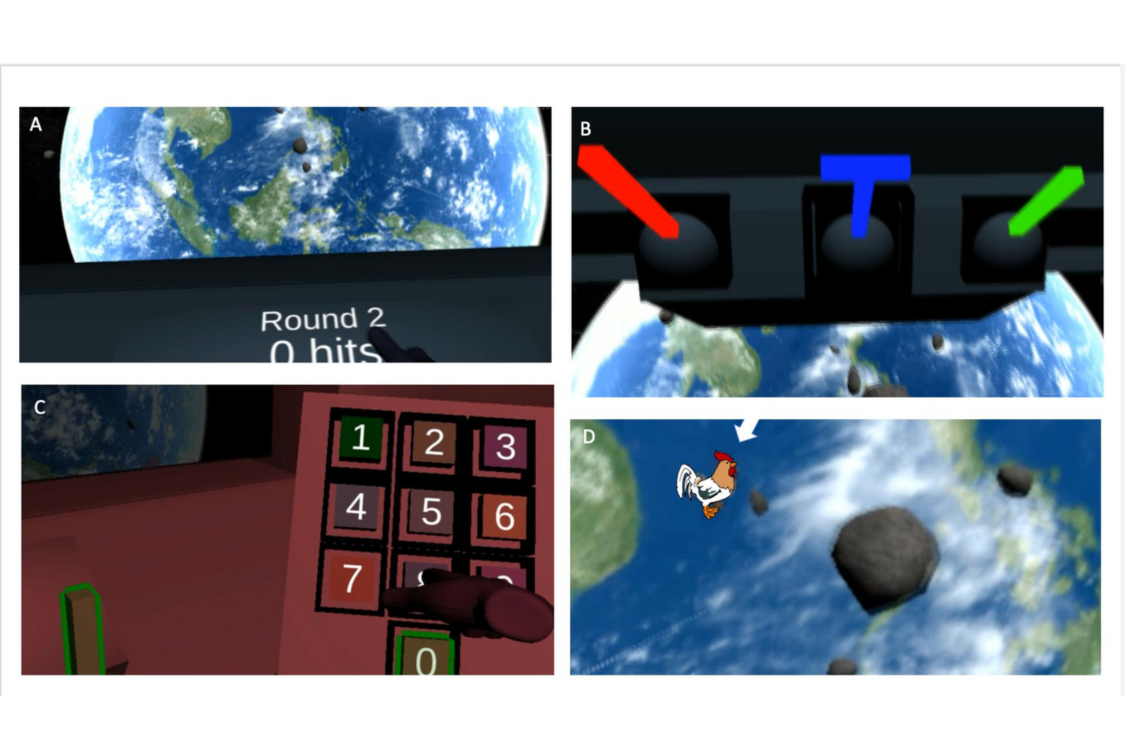

Supplement: Multimedia Appendix 1 [file formative_v8i1e60674_app1.png]
