# Supplementary material for: Evaluating a Virtual Reality Game to Enhance Teen Distracted Driving Education: Mixed Methods Pilot Study
Source: JMIR Form Res. 2024 Nov 26;8:e60674. doi: 10.2196/60674 (PMC11632282; doi:10.2196/60674)
Supplement: Multimedia Appendix 3 [file formative_v8i1e60674_app3.doc]

**Semistructured focus group guide**

**Guidelines**

- No right or wrong answers, only differing points of view
- Remember to keep what’s said here confidential
- We're tape recording, so one person speaking at a time
- You don't need to agree with others, but you must listen respectfully as others share their views
- My role as moderator will be to guide the discussion
- Feel free to talk to each other

**Moderator Prompts**

**We’d like to get a couple of your thoughts on the program you just experienced.**

What are your general reactions to the programming?

         How would you describe the game and discussion to someone who wasn’t present?

Take a moment to jot down 3 things you learned from the program.

[each share with group]

How did today’s activities change how likely you are to text and drive?

**Now we’ll talk about the VR game in particular.**

How well did the VR game make the connection to distracted driving?

After learning about (shared attention, task switching, and inattention blindness), how well did the game play demonstrate this? [Remind as needed what these mean]

How well did the program demonstrate the risks of texting and driving?

         To put another way, how well did the VR game make the connection between distracted driving and crash risk?

What are the features of the VR game that made a difference? What influences that?

What would you add or take away? What influences that?

How did the gameplay haptic feedback and sounds help or not?

What needs improvement?

What was different for you during active play versus when watching others play?

Do you feel like just watching would have been informative?

**Now let’s talk about the post-VR gaming discussion.**

What did you like best about the materials and the discussion?

What needs improvement?

How did the post-game discussion influence your thoughts on the dangers of texting and driving?

         What did you find most useful about the post-VR discussion? Least useful?

What did you think of the strategies presented to not text and drive?

         What are you going to do to avoid texting and driving?

What are the features of the post-game discussion that made a difference? What influences that?

What would you add or take away? What influences that?

**Wrap up**

If you scored the usefulness of the programming , from 1 to 10 with 1 being ___ and 10 being _, what score would you give it? [have everyone write down number]

         Share and explain [ask everyone]

Of all the things we’ve talked about, what was most important? [ask everyone]

We discussed X topic at length, but we didn’t hear much about Y. Any other thoughts on Y?

Is there a specific topic we want to circle back to from this discussion to add or expand on?

**Table S1**. Exploratory full pre- and postregression mixed effect hierarchical modeling of theory of planned behavior concepts testing treatment by time effect.

|  | | ß (95% CI) | SE | | | t | | *P* value |
| --- | --- | --- | --- | --- | --- | --- | --- | --- |
| ***Perceived behavioral control*** | | | | | | | | |
| **I am confident that I could text while driving and still drive safely.** | | | | | | | | |
|  | Intervention (yes) | 0.05 (-1.03, 1.12) | | 0.53 | 0.09 | | 0.93 | |
|  | Time (Follow-up) | -0.86 (-1.79, 0.08) | | 0.46 | -1.86 | | 0.07 | |
|  | Intervention*Time | 0.11 (-1, 1.23) | | 0.55 | 0.20 | | 0.84 | |
|  | Read texts ever | 0.03 (-1.01, 1.07) | | 0.52 | 0.06 | | 0.96 | |
|  | Send texts ever | 0.73 (-0.2, 1.65) | | 0.46 | 1.59 | | 0.12 | |
|  | Intercept | 2.03 (0.94, 3.11) | | 0.54 | 3.77 | | <.01 | |
|  |  |  | |  |  | |  | |
| **It would be easy for me to text while driving in the next week.** | | | | | | | | |
|  | Intervention (yes) | 0.5 (-0.5, 1.5) | | 0.49 | 1.01 | | 0.32 | |
|  | Time (Follow-up) | -0.57 (-1.71, 0.56) | | 0.56 | -1.02 | | 0.32 | |
|  | Intervention*Time | 0.12 (-1.21, 1.45) | | 0.66 | 0.18 | | 0.86 | |
|  | Read texts ever | 0.77 (-0.15, 1.7) | | 0.46 | 1.69 | | 0.10 | |
|  | Send texts ever | 0.72 (-0.1, 1.54) | | 0.41 | 1.78 | | 0.08 | |
|  | Intercept | 1.11 (0.11, 2.1) | | 0.49 | 2.25 | | 0.03 | |
|  |  |  | |  |  | |  | |
| **Perceived behavioral control sum** | | | | | | | | |
|  | Intervention (yes) | 0.56 (-1.3, 2.42) | | 0.92 | 0.61 | | 0.55 | |
|  | Time (Follow-up) | -1.43 (-3.09, 0.23) | | 0.82 | -1.74 | | 0.09 | |
|  | Intervention*Time | 0.21 (-1.77, 2.19) | | 0.98 | 0.21 | | 0.83 | |
|  | Read texts ever | 0.81 (-0.97, 2.58) | | 0.88 | 0.92 | | 0.36 | |
|  | Send texts ever | 1.48 (-0.1, 3.05) | | 0.78 | 1.89 | | 0.07 | |
|  | Intercept | 3.11 (1.25, 4.98) | | 0.92 | 3.37 | | <.01 | |
|  |  |  | |  |  | |  | |

| ***General texting while driving attitude*** | | | | | |
| --- | --- | --- | --- | --- | --- |
| **Texting while driving can be dangerous, but I am going to do it anyway.** | | | | | |
|  | Intervention (yes) | 1.03 (0.12, 1.93) | 0.45 | 2.29 | **0.03** |
|  | Time (Follow-up) | -0.14 (-0.88, 0.6) | 0.37 | -0.39 | 0.70 |
|  | Intervention*Time | -0.21 (-1.09, 0.66) | 0.43 | -0.49 | 0.63 |
|  | Read texts ever | 0.23 (-0.69, 1.15) | 0.45 | 0.51 | 0.61 |
|  | Send texts ever | 1.02 (0.2, 1.84) | 0.40 | 2.52 | **0.02** |
|  | Intercept | 0.5 (-0.43, 1.43) | 0.46 | 1.09 | 0.28 |
|  |  |  |  |  |  |
| **It is my business if I want to text while driving.** | | | | | |
|  | Intervention (yes) | -0.58 (-1.4, 0.23) | 0.40 | -1.45 | 0.16 |
|  | Time (Follow-up) | -0.57 (-1.35, 0.21) | 0.39 | -1.48 | 0.15 |
|  | Intervention*Time | 0.56 (-0.34, 1.47) | 0.45 | 1.25 | 0.22 |
|  | Read texts ever | -0.48 (-1.3, 0.35) | 0.41 | -1.17 | 0.25 |
|  | Send texts ever | 0.72 (-0.02, 1.45) | 0.36 | 1.98 | 0.06 |
|  | Intercept | 1.9 (1.07, 2.73) | 0.41 | 4.61 | <.01 |
|  |  |  |  |  |  |
| **Attitude sum** | | | | | |
|  | Intervention (yes) | 0.47 (-0.95, 1.89) | 0.70 | 0.66 | 0.51 |
|  | Time (Follow-up) | -0.71 (-1.67, 0.24) | 0.47 | -1.51 | 0.14 |
|  | Intervention*Time | 0.36 (-0.78, 1.51) | 0.57 | 0.64 | 0.53 |
|  | Read texts ever | -0.2 (-1.67, 1.27) | 0.73 | -0.28 | 0.79 |
|  | Send texts ever | 1.73 (0.42, 3.03) | 0.65 | 2.67 | **0.01** |
|  | Intercept | 2.37 (0.91, 3.83) | 0.72 | 3.27 | <.01 |
|  |  |  |  |  |  |

| ***Subjective norms*** | | | | | |
| --- | --- | --- | --- | --- | --- |
| **Most people important to me would want me to text while driving in the next week.** | | | | | |
|  | Intervention (yes) | 0.54 (0.24, 0.84) | 0.15 | 3.66 | **<.01** |
|  | Time (Follow-up) | 0 (0, 0) | 0.00 | 0.00 | 1.00 |
|  | Intervention*Time | 0 (0, 0) | 0.00 | 0.00 | 1.00 |
|  | Read texts ever | 0.46 (0.12, 0.8) | 0.17 | 2.76 | **0.01** |
|  | Send texts ever | 0.19 (-0.11, 0.49) | 0.15 | 1.28 | 0.21 |
|  | Intercept | 0.47 (0.15, 0.79) | 0.16 | 2.97 | <.01 |
|  |  |  |  |  |  |
| **Most people important to me would approve of me texting while driving in the next week.** | | | | | |
|  | Intervention (yes) | 0.24 (-0.24, 0.71) | 0.23 | 1.01 | 0.32 |
|  | Time (Follow-up) | -0.14 (-0.57, 0.28) | 0.21 | -0.68 | 0.50 |
|  | Intervention*Time | 0.02 (-0.48, 0.53) | 0.25 | 0.10 | 0.92 |
|  | Read texts ever | -0.01 (-0.46, 0.44) | 0.22 | -0.06 | 0.95 |
|  | Send texts ever | 0.36 (-0.04, 0.76) | 0.20 | 1.82 | 0.08 |
|  | Intercept | 0.9 (0.42, 1.37) | 0.24 | 3.81 | <.01 |
|  |  |  |  |  |  |
| **Most people important to me would think that I should text while driving in the next week.** | | | | | |
|  | Intervention (yes) | 0.47 (0.06, 0.88) | 0.20 | 2.32 | **0.03** |
|  | Time (Follow-up) | 0 (-0.26, 0.26) | 0.13 | 0.00 | 1.00 |
|  | Intervention*Time | -0.19 (-0.49, 0.12) | 0.15 | -1.21 | 0.23 |
|  | Read texts ever | 0.19 (-0.16, 0.55) | 0.18 | 1.10 | 0.28 |
|  | Send texts ever | 0.27 (-0.05, 0.58) | 0.16 | 1.73 | 0.09 |
|  | Intercept | 0.64 (0.24, 1.05) | 0.20 | 3.21 | <.01 |
|  |  |  |  |  |  |
| **Subjective norms sum** | | | | | |
|  | Intervention (yes) | 1.25 (0.01, 2.49) | 0.61 | 2.04 | **0.05** |
|  | Time (Follow-up) | -0.14 (-0.73, 0.44) | 0.29 | -0.50 | 0.62 |
|  | Intervention*Time | -0.17 (-0.86, 0.53) | 0.35 | -0.48 | 0.63 |
|  | Read texts ever | 0.64 (-0.6, 1.88) | 0.61 | 1.04 | 0.30 |
|  | Send texts ever | 0.83 (-0.27, 1.92) | 0.54 | 1.53 | 0.13 |
|  | Intercept | 2 (0.74, 3.27) | 0.63 | 3.19 | <.01 |
|  |  |  |  |  |  |
| ***Perceived disadvantages of abstention*** | | | | | |
| **I could not check with someone if I got lost or forgot something.** | | | | | |
|  | Intervention (yes) | 0.54 (-0.61, 1.69) | 0.57 | 0.96 | 0.35 |
|  | Time (Follow-up) | 0.14 (-0.81, 1.1) | 0.47 | 0.30 | 0.76 |
|  | Intervention*Time | -0.62 (-1.76, 0.52) | 0.57 | -1.09 | 0.28 |
|  | Read texts ever | 0.77 (-0.27, 1.8) | 0.51 | 1.49 | 0.14 |
|  | Send texts ever | -0.2 (-1.12, 0.71) | 0.45 | -0.45 | 0.66 |
|  | Intercept | 2.34 (1.2, 3.49) | 0.57 | 4.13 | <.01 |
|  |  |  |  |  |  |
| **I would not be able to tell people where I am or when I will arrive.** | | | | | |
|  | Intervention (yes) | 0.38 (-0.83, 1.59) | 0.60 | 0.63 | 0.53 |
|  | Time (Follow-up) | -0.29 (-1.22, 0.65) | 0.46 | -0.62 | 0.54 |
|  | Intervention*Time | -0.04 (-1.15, 1.08) | 0.55 | -0.07 | 0.95 |
|  | Read texts ever | 1.75 (0.53, 2.96) | 0.60 | 2.90 | **0.01** |
|  | Send texts ever | -0.44 (-1.52, 0.65) | 0.54 | -0.81 | 0.42 |
|  | Intercept | 2.1 (0.86, 3.34) | 0.61 | 3.43 | <.01 |
|  |  |  |  |  |  |
| **My mom or dad could not call me when they need me.** | | | | | |
|  | Intervention (yes) | 0.32 (-1.04, 1.69) | 0.68 | 0.48 | 0.63 |
|  | Time (Follow-up) | -0.57 (-1.7, 0.55) | 0.56 | -1.03 | 0.31 |
|  | Intervention*Time | 0.5 (-0.84, 1.85) | 0.67 | 0.75 | 0.46 |
|  | Read texts ever | 1.07 (-0.27, 2.41) | 0.66 | 1.61 | 0.12 |
|  | Send texts ever | 0.03 (-1.16, 1.23) | 0.59 | 0.05 | 0.96 |
|  | Intercept | 2.06 (0.68, 3.45) | 0.69 | 3.01 | <.01 |
|  |  |  |  |  |  |
| **I would be different from all my friends.** | | | | | |
|  | Intervention (yes) | 0.85 (-0.3, 1.99) | 0.57 | 1.50 | 0.14 |
|  | Time (Follow-up) | 0.43 (-0.99, 1.84) | 0.70 | 0.61 | 0.54 |
|  | Intervention*Time | -0.44 (-2.05, 1.18) | 0.80 | -0.55 | 0.59 |
|  | Read texts ever | 0.89 (-0.3, 2.07) | 0.59 | 1.51 | 0.14 |
|  | Send texts ever | 0.16 (-0.88, 1.21) | 0.52 | 0.31 | 0.76 |
|  | Intercept | 0.84 (-0.34, 2.02) | 0.58 | 1.43 | 0.16 |
|  |  |  |  |  |  |
| **Disadvantages sum** | | | | | |
|  | Intervention (yes) | 2.31 (-2.15, 6.77) | 2.21 | 1.04 | 0.30 |
|  | Time (Follow-up) | -0.29 (-3.05, 2.48) | 1.37 | -0.21 | 0.84 |
|  | Intervention*Time | -0.66 (-3.96, 2.65) | 1.64 | -0.40 | 0.69 |
|  | Read texts ever | 4.68 (1.05, 8.3) | 1.79 | 2.61 | **0.01** |
|  | Send texts ever | -0.29 (-3.48, 2.89) | 1.58 | -0.18 | 0.86 |
|  | Intercept | 7.06 (2.7, 11.41) | 2.15 | 3.27 | <.01 |
|  |  |  |  |  |  |
| ***Perceived advantages of abstention*** | | | | | |
| **I would be able to pay better attention to my driving.** | | | | | |
|  | Intervention (yes) | -0.44 (-1.43, 0.54) | 0.49 | -0.91 | 0.37 |
|  | Time (Follow-up) | -0.57 (-1.23, 0.09) | 0.33 | -1.75 | 0.09 |
|  | Intervention*Time | 0.57 (-0.22, 1.36) | 0.39 | 1.46 | 0.15 |
|  | Read texts ever | -0.16 (-1.26, 0.95) | 0.55 | -0.29 | 0.78 |
|  | Send texts ever | 0.02 (-0.96, 1) | 0.49 | 0.04 | 0.97 |
|  | Intercept | 5.12 (4.08, 6.16) | 0.52 | 9.92 | <.01 |
|  |  |  |  |  |  |
| **I would be less likely to get into an accident.** | | | | | |
|  | Intervention (yes) | -0.21 (-0.58, 0.17) | 0.19 | -1.11 | 0.28 |
|  | Time (Follow-up) | -0.71 (-1.63, 0.2) | 0.45 | -1.57 | 0.12 |
|  | Intervention*Time | 0.47 (-0.63, 1.56) | 0.54 | 0.86 | 0.40 |
|  | Read texts ever | 0.08 (-0.22, 0.39) | 0.15 | 0.53 | 0.60 |
|  | Send texts ever | -0.34 (-0.61, -0.07) | 0.13 | -2.55 | **0.02** |
|  | Intercept | 5.17 (4.8, 5.54) | 0.18 | 28.36 | <.01 |
|  |  |  |  |  |  |
| **I would be following the law.** | | | | | |
|  | Intervention (yes) | 0.1 (-0.23, 0.42) | 0.16 | 0.60 | 0.55 |
|  | Time (Follow-up) | -0.43 (-1.36, 0.51) | 0.46 | -0.93 | 0.36 |
|  | Intervention*Time | 0.11 (-1.01, 1.23) | 0.55 | 0.20 | 0.84 |
|  | Read texts ever | 0.03 (-0.33, 0.39) | 0.18 | 0.19 | 0.85 |
|  | Send texts ever | -0.02 (-0.34, 0.3) | 0.16 | -0.12 | 0.91 |
|  | Intercept | 4.84 (4.5, 5.19) | 0.17 | 28.36 | <.01 |
|  |  |  |  |  |  |
| **I would make my parents happy.** | | | | | |
|  | Intervention (yes) | -0.09 (-0.47, 0.3) | 0.19 | -0.45 | 0.66 |
|  | Time (Follow-up) | -0.43 (-1.5, 0.64) | 0.53 | -0.81 | 0.42 |
|  | Intervention*Time | 0.12 (-1.14, 1.39) | 0.63 | 0.20 | 0.84 |
|  | Read texts ever | -0.14 (-0.56, 0.28) | 0.21 | -0.66 | 0.52 |
|  | Send texts ever | -0.06 (-0.43, 0.31) | 0.18 | -0.33 | 0.75 |
|  | Intercept | 5.02 (4.61, 5.42) | 0.20 | 25.15 | <.01 |
|  |  |  |  |  |  |
| **I would be calmer when I drive because I would not have conversations that might upset me.** | | | | | |
|  | Intervention (yes) | -1.23 (-1.99, -0.46) | 0.38 | -3.24 | **<.01** |
|  | Time (Follow-up) | -0.14 (-0.93, 0.65) | 0.39 | -0.36 | 0.72 |
|  | Intervention*Time | 0.02 (-0.93, 0.97) | 0.47 | 0.03 | 0.97 |
|  | Read texts ever | -1.13 (-1.99, -0.28) | 0.43 | -2.67 | **0.01** |
|  | Send texts ever | -0.52 (-1.29, 0.24) | 0.38 | -1.38 | 0.18 |
|  | Intercept | 6.06 (5.25, 6.87) | 0.40 | 15.09 | <.01 |
|  |  |  |  |  |  |
| **I would not get a ticket.** | | | | | |
|  | Intervention (yes) | -0.28 (-0.93, 0.37) | 0.32 | -0.86 | 0.39 |
|  | Time (Follow-up) | -0.29 (-1.13, 0.56) | 0.42 | -0.68 | 0.50 |
|  | Intervention*Time | 0.09 (-0.92, 1.11) | 0.50 | 0.18 | 0.86 |
|  | Read texts ever | -0.17 (-0.84, 0.5) | 0.33 | -0.52 | 0.61 |
|  | Send texts ever | -0.05 (-0.65, 0.54) | 0.29 | -0.18 | 0.86 |
|  | Intercept | 5.04 (4.37, 5.71) | 0.33 | 15.19 | 0.00 |
|  |  |  |  |  |  |
| **My friends would think I was responsible.** | | | | | |
|  | Intervention (yes) | -0.56 (-1.14, 0.01) | 0.28 | -1.98 | 0.05 |
|  | Time (Follow-up) | 0.14 (-0.85, 1.13) | 0.49 | 0.29 | 0.77 |
|  | Intervention*Time | -0.5 (-1.66, 0.66) | 0.57 | -0.87 | 0.39 |
|  | Read texts ever | -0.93 (-1.58, -0.28) | 0.32 | -2.89 | **0.01** |
|  | Send texts ever | -0.15 (-0.72, 0.43) | 0.29 | -0.52 | 0.61 |
|  | Intercept | 5.47 (4.86, 6.08) | 0.30 | 18.11 | <.01 |
|  |  |  |  |  |  |
| **Advantage sum** | | | | | |
|  | Intervention (yes) | -2.76 (-5.24, -0.29) | 1.22 | -2.25 | **0.03** |
|  | Time (Follow-up) | -2.43 (-7.56, 2.7) | 2.54 | -0.96 | 0.34 |
|  | Intervention*Time | 0.87 (-5.28, 7.01) | 3.04 | 0.28 | 0.78 |
|  | Read texts ever | -2.5 (-5.29, 0.29) | 1.38 | -1.81 | 0.08 |
|  | Send texts ever | -1.12 (-3.6, 1.36) | 1.23 | -0.91 | 0.37 |
|  | Intercept | 36.8 (34.17, 39.42) | 1.30 | 28.32 | <.01 |
|  |  |  |  |  |  |

| **Intentions** | | | | | |
| --- | --- | --- | --- | --- | --- |
| **I intend to text while driving in the next week.** | | | | | |
|  | Intervention (yes) | 0.84 (0.34, 1.33) | 0.24 | 3.43 | **<.01** |
|  | Time (Follow-up) | 0 (-0.37, 0.37) | 0.18 | 0.00 | 1.00 |
|  | Intervention*Time | -0.24 (-0.68, 0.2) | 0.22 | -1.09 | 0.28 |
|  | Read texts ever | 0.43 (0, 0.87) | 0.22 | 2.00 | 0.05 |
|  | Send texts ever | 0.58 (0.2, 0.97) | 0.19 | 3.05 | **<.01** |
|  | Intercept | 0.36 (-0.13, 0.85) | 0.24 | 1.48 | 0.15 |
|  |  |  |  |  |  |
| **It is likely that I will text while driving in the next week.** | | | | | |
|  | Intervention (yes) | 0.97 (0.09, 1.84) | 0.43 | 2.23 | **0.03** |
|  | Time (Follow-up) | 0.29 (-0.29, 0.86) | 0.28 | 1.01 | 0.32 |
|  | Intervention*Time | -0.46 (-1.14, 0.22) | 0.34 | -1.36 | 0.18 |
|  | Read texts ever | 0.94 (0.17, 1.71) | 0.38 | 2.48 | **0.02** |
|  | Send texts ever | 0.33 (-0.35, 1.01) | 0.33 | 0.99 | 0.33 |
|  | Intercept | 0.24 (-0.62, 1.11) | 0.43 | 0.57 | 0.57 |
|  |  |  |  |  |  |
| **I am willing to text while driving in the next week.** | | | | | |
|  | Intervention (yes) | 1.05 (0.25, 1.85) | 0.40 | 2.65 | **0.01** |
|  | Time (Follow-up) | 0 (-0.69, 0.69) | 0.34 | 0.00 | 1.00 |
|  | Intervention*Time | -0.24 (-1.06, 0.58) | 0.41 | -0.59 | 0.56 |
|  | Read texts ever | 0.74 (-0.13, 1.61) | 0.43 | 1.71 | 0.09 |
|  | Send texts ever | 1 (0.22, 1.78) | 0.38 | 2.60 | **0.01** |
|  | Intercept | 0.36 (-0.48, 1.2) | 0.42 | 0.88 | 0.39 |
|  |  |  |  |  |  |
| **Intentions sum** | | | | | |
|  | Intervention (yes) | 2.79 (0.91, 4.68) | 0.93 | 2.99 | **0.01** |
|  | Time (Follow-up) | 0.29 (-1.11, 1.68) | 0.69 | 0.41 | 0.68 |
|  | Intervention*Time | -0.95 (-2.62, 0.72) | 0.83 | -1.14 | 0.26 |
|  | Read texts ever | 2.1 (0.17, 4.03) | 0.96 | 2.20 | **0.03** |
|  | Send texts ever | 1.77 (0.06, 3.49) | 0.85 | 2.08 | **0.04** |
|  | Intercept | 1.07 (-0.86, 3.01) | 0.96 | 1.12 | 0.27 |

**Table S2**. Exploratory pre- and postregression models of theory of planned behavior concepts by time effect only.

|  | | ß (95% CI) | SE | t | *P* value |
| --- | --- | --- | --- | --- | --- |
| ***Perceived behavioral control*** | | | | | |
| **I am confident that I could text while driving and still drive safely.** | | | | | |
|  | Time (Follow-up) | -0.78 (-1.37, -0.19) | 0.29 | -2.66 | **.01** |
|  | Read texts ever | -0.02 (-0.81, 0.76) | 0.39 | -0.06 | .95 |
|  | Send texts ever | 0.73 (-0.06, 1.51) | 0.39 | 1.86 | .07 |
| **It would be easy for me to text while driving in the next week.** | | | | | |
|  | Time (Follow-up) | -0.50 (-1.07, 0.08) | 0.29 | -1.74 | .09 |
|  | Read texts ever | 0.49 (-0.28, 1.25) | 0.38 | 1.28 | .21 |
|  | Send texts ever | 0.74 (-0.03, 1.5) | 0.38 | 1.93 | .06 |
|  | Intercept | 1.57 (1.09, 2.06) | 0.24 | 6.58 | <.01 |
| **Perceived behavioral control sum** | | | | | |
|  | Time (Follow-up) | -1.28 (-2.31, -0.24) | 0.51 | -2.49 | **.02** |
|  | Read texts ever | 0.46 (-0.92, 1.84) | 0.68 | 0.68 | .50 |
|  | Send texts ever | 1.46 (0.08, 2.84) | 0.68 | 2.14 | **.04** |
|  | Intercept | 3.66 (2.79, 4.52) | 0.43 | 8.50 | <.01 |
| ***General texting while driving attitude*** | | | | | |
| **Texting while driving can be dangerous, but I am going to do it anyway.** | | | | | |
|  | Time (Follow-up) | -0.29 (-0.82, 0.25) | 0.27 | -1.07 | .29 |
|  | Read texts ever | -0.24 (-0.96, 0.47) | 0.35 | -0.69 | .49 |
|  | Send texts ever | 1.01 (0.29, 1.72) | 0.35 | 2.84 | **.007** |
|  | Intercept | 1.43 (0.98, 1.88) | 0.22 | 6.43 | <.01 |

| **It is my business if I want to text while driving.** | | | | | |
| --- | --- | --- | --- | --- | --- |
|  | Time (Follow-up) | -0.16 (-0.61, 0.3) | 0.23 | -0.69 | .50 |
|  | Read texts ever | -0.30 (-0.91, 0.31) | 0.30 | -0.99 | .33 |
|  | Send texts ever | 0.70 (0.09, 1.31) | 0.30 | 2.32 | **.03** |
|  | Intercept | 1.42 (1.03, 1.8) | 0.19 | 7.44 | <.01 |
| **Attitude sum** | | | | | |
|  | Time (Follow-up) | -0.44 (-1.21, 0.33) | 0.38 | -1.15 | .26 |
|  | Read texts ever | -0.54 (-1.57, 0.49) | 0.51 | -1.06 | .29 |
|  | Send texts ever | 1.71 (0.68, 2.74) | 0.51 | 3.34 | **.002** |
|  | Intercept | 2.85 (2.2, 3.5) | 0.32 | 8.86 | <.01 |
| ***Subjective norms*** | | | | | |
| **Most people important to me would want me to text while driving in the next week.** | | | | | |
|  | Time (Follow-up) | 0.00 (-0.25, 0.25) | 0.13 | -0.01 | .99 |
|  | Read texts ever | 0.18 (-0.16, 0.52) | 0.17 | 1.08 | .29 |
|  | Send texts ever | 0.18 (-0.16, 0.52) | 0.17 | 1.08 | .29 |
|  | Intercept | 0.97 (0.76, 1.19) | 0.11 | 9.23 | <.01 |
| **Most people important to me would approve of me texting while driving in the next week.** | | | | | |
|  | Time (Follow-up) | -0.13 (-0.39, 0.14) | 0.13 | -0.95 | .35 |
|  | Read texts ever | -0.14 (-0.5, 0.21) | 0.18 | -0.81 | .43 |
|  | Send texts ever | 0.36 (0, 0.71) | 0.18 | 2.03 | **.049** |
|  | Intercept | 1.12 (0.89, 1.34) | 0.11 | 10.07 | <.01 |
| **Most people important to me would think that I should text while driving in the next week.** | | | | | |
|  | Time (Follow-up) | -0.13 (-0.34, 0.08) | 0.10 | -1.22 | .23 |
|  | Read texts ever | 0.00 (-0.28, 0.28) | 0.14 | 0.01 | .99 |
|  | Send texts ever | 0.25 (-0.03, 0.53) | 0.14 | 1.80 | .08 |
|  | Intercept | 1.06 (0.88, 1.24) | 0.09 | 12.05 | <.01 |
| **Subjective norms sum** | | | | | |
|  | Time (Follow-up) | -0.25 (-0.92, 0.41) | 0.33 | -0.77 | .44 |
|  | Read texts ever | 0.04 (-0.84, 0.93) | 0.44 | 0.09 | .93 |
|  | Send texts ever | 0.79 (-0.09, 1.68) | 0.44 | 1.80 | .08 |
|  | Intercept | 3.15 (2.6, 3.71) | 0.28 | 11.41 | <.01 |
| ***Perceived disadvantages of abstention*** | | | | | |
| **I could not check with someone if I got lost or forgot something.** | | | | | |
|  | Time (Follow-up) | -0.27 (-0.9, 0.35) | 0.31 | -0.88 | .38 |
|  | Read texts ever | 0.79 (-0.05, 1.62) | 0.41 | 1.90 | .06 |
|  | Send texts ever | -0.21 (-1.05, 0.62) | 0.41 | -0.51 | .61 |
|  | Intercept | 2.72 (2.19, 3.24) | 0.26 | 10.43 | <.01 |
| **I would not be able to tell people where I am or when I will arrive.** | | | | | |
|  | Time (Follow-up) | -0.28 (-0.94, 0.37) | 0.33 | -0.87 | .39 |
|  | Read texts ever | 1.54 (0.67, 2.42) | 0.44 | 3.55 | **.001** |
|  | Send texts ever | -0.46 (-1.33, 0.42) | 0.44 | -1.05 | .30 |
|  | Intercept | 2.46 (1.91, 3.02) | 0.27 | 8.99 | <.01 |
| **My mom or dad could not call me when they need me.** | | | | | |
|  | Time (Follow-up) | -0.20 (-0.96, 0.56) | 0.38 | -0.53 | .60 |
|  | Read texts ever | 0.76 (-0.25, 1.77) | 0.50 | 1.52 | .14 |
|  | Send texts ever | 0.01 (-1, 1.02) | 0.50 | 0.02 | .98 |
|  | Intercept | 2.43 (1.79, 3.06) | 0.32 | 7.70 | <.01 |
| **I would be different from all my friends.** | | | | | |
|  | Time (Follow-up) | 0.11 (-0.54, 0.77) | 0.32 | 0.35 | .73 |
|  | Read texts ever | 0.43 (-0.44, 1.3) | 0.43 | 0.99 | .33 |
|  | Send texts ever | 0.18 (-0.69, 1.05) | 0.43 | 0.41 | .68 |
|  | Intercept | 1.62 (1.07, 2.17) | 0.27 | 5.97 | <.01 |
| ***Disadvantages sum*** | | | | | |
|  | Time (Follow-up) | -0.64 (-2.67, 1.38) | 1.01 | -0.64 | .53 |
|  | Read texts ever | 3.52 (0.82, 6.22) | 1.34 | 2.63 | **.01** |
|  | Send texts ever | -0.48 (-3.18, 2.22) | 1.34 | -0.36 | .72 |
|  | Intercept | 9.23 (7.53, 10.93) | 0.84 | 10.95 | <.01 |
| ***Perceived advantages of abstention*** | | | | | |
| **I would be able to pay better attention to my driving.** | | | | | |
|  | Time (Follow-up) | -0.18 (-0.79, 0.43) | 0.30 | -0.61 | .55 |
|  | Read texts ever | 0.09 (-0.72, 0.9) | 0.40 | 0.23 | .82 |
|  | Send texts ever | -0.16 (-0.97, 0.65) | 0.40 | -0.40 | .69 |
|  | Intercept | 4.78 (4.27, 5.29) | 0.25 | 18.87 | <.01 |
| **I would be less likely to get into an accident.** | | | | | |
|  | Time (Follow-up) | -0.39 (-0.89, 0.11) | 0.25 | -1.58 | .12 |
|  | Read texts ever | 0.03 (-0.64, 0.69) | 0.33 | 0.08 | .94 |
|  | Send texts ever | -0.22 (-0.89, 0.44) | 0.33 | -0.68 | .50 |
|  | Intercept | 5.00 (4.58, 5.42) | 0.21 | 24.02 | <.01 |
| **I would be following the law.** | | | | | |
|  | Time (Follow-up) | -0.35 (-0.89, 0.11) | 0.25 | -1.40 | 0.17 |
|  | Read texts ever | -0.04 (-0.64, 0.69) | 0.33 | -0.13 | 0.90 |
|  | Send texts ever | -0.04 (-0.89, 0.44) | 0.33 | -0.13 | 0.90 |
|  | Intercept | 4.95 (4.58, 5.42) | 0.21 | 23.66 | <.01 |
| **I would make my parents happy.** | | | | | |
|  | Time (Follow-up) | -0.35 (-0.86, 0.16) | 0.25 | -1.39 | .17 |
|  | Read texts ever | -0.20 (-0.88, 0.47) | 0.34 | -0.60 | .55 |
|  | Send texts ever | 0.05 (-0.63, 0.72) | 0.34 | 0.14 | .89 |
|  | Intercept | 4.94 (4.51, 5.37) | 0.21 | 23.38 | <.01 |
| **I would be calmer when I drive because I would not have conversations that might upset me.** | | | | | |
|  | Time (Follow-up) | -0.14 (-0.74, 0.47) | 0.30 | -0.46 | .65 |
|  | Read texts ever | -0.39 (-1.2, 0.41) | 0.40 | -0.99 | .33 |
|  | Send texts ever | -0.39 (-1.2, 0.41) | 0.40 | -0.99 | .33 |
|  | Intercept | 4.83 (4.32, 5.34) | 0.25 | 19.21 | <.01 |
| **I would not get a ticket.** | | | | | |
|  | Time (Follow-up) | -0.23 (-0.74, 0.28) | 0.25 | -0.91 | .37 |
|  | Read texts ever | 0.01 (-0.66, 0.69) | 0.34 | 0.04 | .97 |
|  | Send texts ever | 0.01 (-0.66, 0.69) | 0.34 | 0.04 | .97 |
|  | Intercept | 4.74 (4.31, 5.17) | 0.21 | 22.35 | <.01 |
| **My friends would think I was responsible.** | | | | | |
|  | Time (Follow-up) | -0.23 (-0.8, 0.35) | 0.28 | -0.79 | .43 |
|  | Read texts ever | -0.47 (-1.23, 0.29) | 0.38 | -1.24 | .22 |
|  | Send texts ever | 0.03 (-0.73, 0.79) | 0.38 | 0.08 | .94 |
|  | Intercept | 4.81 (4.33, 5.29) | 0.24 | 20.16 | <.01 |
| **Advantage sum** | | | | | |
|  | Time (Follow-up) | -1.87 (-5.14, 1.4) | 1.62 | -1.15 | .26 |
|  | Read texts ever | -0.98 (-5.34, 3.38) | 2.16 | -0.45 | .65 |
|  | Send texts ever | -0.73 (-5.09, 3.63) | 2.16 | -0.34 | .74 |
|  | Intercept | 34.04 (31.3, 36.79) | 1.36 | 25.00 | <.01 |
| ***Intentions*** | | | | | |
| **I intend to text while driving in the next week.** | | | | | |
|  | Time (Follow-up) | -0.17 (-0.47, 0.13) | 0.15 | -1.14 | .26 |
|  | Read texts ever | 0.07 (-0.33, 0.47) | 0.20 | 0.35 | .72 |
|  | Send texts ever | 0.57 (0.17, 0.97) | 0.20 | 2.88 | **.006** |
|  | Intercept | 1.11 (0.86, 1.36) | 0.12 | 8.89 | <.01 |
| **It is likely that I will text while driving in the next week.** | | | | | |
|  | Time (Follow-up) | -0.04 (-0.5, 0.42) | 0.23 | -0.18 | .86 |
|  | Read texts ever | 0.58 (-0.03, 1.18) | 0.30 | 1.92 | .06 |
|  | Send texts ever | 0.33 (-0.28, 0.93) | 0.30 | 1.09 | .28 |
|  | Intercept | 1.08 (0.7, 1.46) | 0.19 | 5.71 | <.01 |
| **I am willing to text while driving in the next week.** | | | | | |
|  | Time (Follow-up) | -0.16 (-0.68, 0.37) | 0.26 | -0.60 | .55 |
|  | Read texts ever | 0.26 (-0.43, 0.96) | 0.34 | 0.76 | .45 |
|  | Send texts ever | 0.76 (0.07, 1.46) | 0.34 | 2.21 | **.03** |
|  | Intercept | 1.41 (0.97, 1.84) | 0.22 | 6.48 | <.01 |
| ***Intention sum*** | | | | | |
|  | Time (Follow-up) | -0.37 (-1.52, 0.79) | 0.57 | -0.64 | .53 |
|  | Read texts ever | 0.91 (-0.64, 2.45) | 0.77 | 1.19 | .24 |
|  | Send texts ever | 1.66 (0.11, 3.2) | 0.77 | 2.16 | **.04** |
|  | Intercept | 3.60 (2.62, 4.57) | 0.48 | 7.46 | <.01 |
